# The effects of an online basic life support course on undergraduate nursing students’ learning

**DOI:** 10.5116/ijme.5985.cbce

**Published:** 2017-08-25

**Authors:** Lucia Tobase, Heloisa H.C. Peres, Renan Gianotto-Oliveira, Nicole Smith, Thatiane F. Polastri, Sergio Timerman

**Affiliations:** 1 Personnel Management, Mobile Emergency Care Service, Sao Paulo, Brazil; 2 Department of Professional Counseling, School of Nursing, University of Sao Paulo, Sao Paulo, Brazil; 3 Department of Emergency Medicine, Campinas State University (UNICAMP), Campinas, Brazil; 4 Heart Sarver Center, University of Arizona College of Medicine, Tucson, USA; 5 Laboratory of Cardiovascular Emergencies Training, Heart Institute (InCor), Clinicals Hospital of the Sao Paulo University, Sao Paulo, Brazil

**Keywords:** Basic life support, education, nursing, cardiopulmonary resuscitation

## Abstract

**Objectives:**

To describe learning outcomes of undergraduate nursing students following an online basic life support course (BLS).

**Methods:**

An online BLS course was developed and administered to 94 nursing students. Pre- and post-tests were used to assess theoretical learning. Checklist simulations and feedback devices were used to assess the cardiopulmonary resuscitation (CPR) skills of the 62 students who completed the course.

**Results:**

A paired t-test revealed a significant increase in learning [pre-test (6.4 ± 1.61), post-test (9.3 ± 0.82), p < 0.001]. The increase in the average grade after taking the online course was significant (p<0.001). No learning differences (p=0.475) had been observed between 1st and 2nd year (9.20 ± 1.60), and between 3rd and 4th year (9.67 ± 0.61) students. A CPR simulation was performed after completing the course: students checked for a response (90%), exposed the chest (98%), checked for breathing (97%), called emergency services (76%), requested for a defibrillator (92%), checked for a pulse (77%), positioned their hands properly (87%), performed 30 compressions/cycle (95%), performed compressions of at least 5 cm depth (89%), released the chest (90%), applied two breaths (97%), used the automated external defibrillator (97%), and positioned the pads (100%).

**Conclusions:**

The online course was an effective method for teaching and learning key BLS skills wherein students were able to accurately apply BLS procedures during the CPR simulation. This short-term online training, which likely improves learning and self-efficacy in BLS providers, can be used for the continuing education of health professionals.

## Introduction

Cardiac arrest is a major public health issue and a cause of mortality worldwide. Higher rates of survival have been seen when cardiac arrests are witnessed.^1^^,^^2^ Survival can even be three times higher when cardiac arrests are attended by persons able to provide immediate resuscitation.^2^ However, only a minority of cardiac arrest victims receive potentially lifesaving bystander cardiopulmonary resuscitation (CPR), thus indicating the need for improvements in resuscitation education.^3^

Resuscitation science is very complex and has its own features depending on the country and culture wherein it is applied. Resuscitation education is primarily focused on ensuring widespread and uniform implementation of resuscitation science, during practice by lay and healthcare CPR providers, to achieve the best possible performance of CPR skills. Some examples include improving healthcare professionals’ ability to recognize and respond to patients at risk for cardiac arrest, improving CPR performance (mainly chest compressions), and ensuring continuous quality improvement activities to optimize future performance through targeted education.^3^ Basic life support (BLS) training recommendations in the 2015 American Heart Association (AHA) Guidelines include the use of high-fidelity manikins, simulations, feedback devices, more frequent training, and short online courses as resources for teaching and learning resuscitation skills in continuing education.^3^^-^^5^

Medical education also uses an effective alternative educational system called e-Learning to facilitate skill development, autonomy, cost-effectiveness, decreased instructor burden, standardization, and evaluation of online material by course organizers, while maintaining positive learning outcomes, satisfaction, and confidence levels of the participants.^4^^-^^8^ However, an online course requires careful planning and organization wherein educational objectives must be defined, content relevant to the participant profile selected, and distribution of the workload performed.^9^

Combining technological resources to increase resuscitation education, the aim of this study was to describe learning outcomes among undergraduate nursing students following an online BLS course (e-BLS).

## Methods

### Study design and participants

In this quasi-experimental study, an online BLS course was developed and administered to 1st–4th year nursing students as an educational intervention at a public university in São Paulo, Brazil. This study was approved by the Research Ethics Committee on February 11, 2014.

### Sampling and sample size

**Figure 1:**
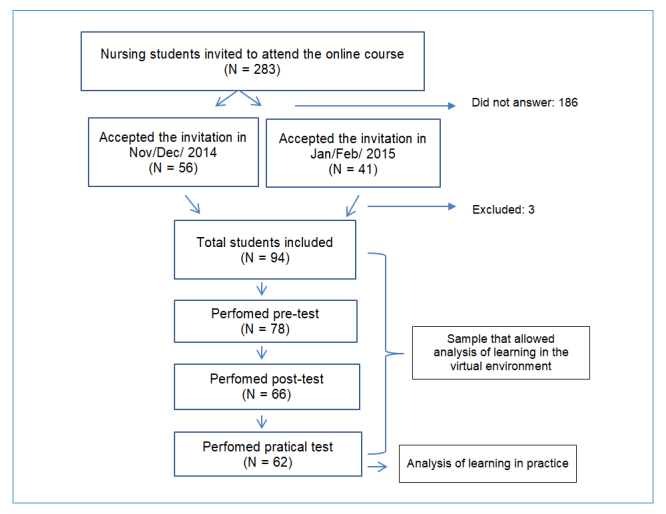
Flow diagram of the participants

Convenience sampling was done by emailing 283 students regularly enrolled in the nursing school. Among them, 97 students agreed to participate in the initial survey. Although 94 students were eligible according to the established criteria, only 62 students completed the online course and participated in all of the required steps shown below (theoretical pre-test, virtual class, theoretical test, and practical test) (Figure 1).

### Data collection

Data was first collected between November 2014 and February 2015 using an electronic form to identify student profiles. Theoretical learning in the virtual environment was evaluated using a 20-question pre- and post-test. Prior to administration, the testing instrument was analyzed by eight nurses specializing in Emergency and Distance Education. After completing the online course, students’ performances in a simulated scenario of cardiorespiratory arrest were directly evaluated based on a 20-item checklist. The students, in pairs, applied the basic life support with automatic external defibrillator (AED), using a simulator with feedback devices.

The quality of compressions was evaluated using a resuscitation manikin and its software. According to the manufacturer, CPR quality is classified by the following scores: 0%–49% (Basic CPR); 50%–74% (Intermediate CPR); and 75%–100% (Advanced CPR).^10^ The total score indicated by the device at the end of the practice is dependent on several sub-scores: Depth of compression, Frequency of compression, Release of the thorax at each compression, Number of compressions/cycle, Position of the hands, Frequency and volume of ventilation, and Flow fraction.^10^

### Procedure

First, we developed an online self-instructional BLS course with a 20 h workload using the ADDIE (Analysis, Design, Development, Implementation, Evaluation) instructional design model.^11^ The definitions of educational objectives and teaching strategies were based on Bloom's Taxonomy and Andragogy.^12^ The online course was implemented using the Moodle platform and evaluated by 12 nurses with experience in Distance Education and Emergency. All students received a login and password to access the virtual environment and were supervised by eight nurses specializing in Distance Education and Emergency.

During the simulation, the skills of the students who completed the study in the virtual environment were evaluated. Students applied the required BLS steps in pairs for 2 min using an automated external defibrillator and a manikin simulator with CPR feedback devices. The students then switched roles for verification of skills in all required procedures. Skills during the practicum were evaluated by two instructors using a printed checklist and electronic feedback devices. Finally, the students evaluated the quality of the online BLS course.

### Data analysis

Means and standard deviations were calculated for pre-, post- and practical test scores. Absolute and relative rates were used for gender, age, and motivation for participation. Variables with non-normal distribution were evaluated using Kendall’s correlation coefficient. Furthermore, t-tests were used to determine differences between the means of pre- and post-test scores, which were used as a parameter for evaluating learning. Analysis of variance (ANOVA) was applied to evaluate theoretical learning. Multiple linear regression was used to assess the association between theoretical learning (dependent variable) and nursing year and prior completion of an emergency course (independent variables). Confidence intervals were set at 95%, while a p-value <0.05 was considered significant. SPSS Statistics version 22.0 for Windows was used for all statistical analyses.

## Results

The study sample consisted of 62 students who completed the entire protocol, among whom 87% were women, the mean age (±SD) was 21.47 (±2.39), 90.3% were 1st and 2nd year students, 9.7% were 3rd and 4th year students, 50% had not participated in previous emergency training, 53.3% had no prior knowledge of BLS, 61.2% were familiar with the Moodle virtual platform, 96.8% were motivated by the practical application of learning, and 69.1% had not participated in a distance education course before.

**Table 1:** Description of pre- and post-test scores by course year

Course year	Test	Mean	SD^*^	CI^**^	Phase	Interaction
					p-value	
1^st^–2^nd^	Pre-test	6.19	1.59	5.76, 6.61	<0.001	0.475
	Post-test	9.20	1.60	8.98, 9.41		
3^rd^–4^th^	Pre-test	7.17	0.83	5.87, 8.46		
	Post-test	9.67	0.61	9.00,10.33		

^*^Standard Deviation; ^**^CI: Confidence Interval

The course was not for approval or disapproval purposes. Furthermore, we used the difference between the mean of post- and pre-test scores of the written test to evaluate learning. The written exam included 20 multiple-choice questions with a score parameter of 8.4. The paired t-test was used to compare the mean scores of the pre-test (6.4 ± 1.61) and post-test (9.3 ± 0.82, p<0.001), which showed notable improvements and significant differences.

Differences in the mean scores of the pre- and post-tests were analyzed using two groups in relation to course year, comparing students from the 1st–2nd and 3rd–4th years. ANOVA showed significant differences in the pre-test scores between the 56 students from the first two years (6.19 ± 1.59) and the group of 6 students from the last two years (7.17 ± 0.83). The analysis indicated no difference (p= 0.475) in learning between 1st–2nd year students (9.20 ± 1.60) and 3rd–4th year students (9.67 ± 0.61). However, a significant increase in the average scores after taking the online course was observed (p<0.001), regardless of the course year of the students (Table 1).

To identify the variables associated with learning, an adjusted multiple linear regression model was used. A significant association between learning and course year was found when the students participated in an emergency course before the online course. Moreover, inversely proportional associations and higher gains were seen for students in their early years (r = −0.542, p = 0.015) or those who did not previously participate in an emergency course (Table 2).

**Table 2:** Results of the multiple linear regression model for course year, emergency attendance, and theoretical learning

Variables	Coefficient	Standard error	p-value
Course year	- 0.542	0.215	0.015
Participation in emergency course	- 0.903	0.437	0.044

R^2^= 0.253

After the online BLS course, the participants performed a CPR simulation (practical test), which had a mean score ± SD of 9.1 ± 0.95 (score from 0 to 10).

Due to technical reasons, we were unable to record the data of 10 participants who used feedback devices. Therefore, the following results refer to only 52 participants. According to the checklist records, following 90% of the participants checked for responsiveness, 97% checked for breathing, 76% activated the emergency response system, 92% requested for a defibrillator, 77% checked for a carotid pulse, 87% used proper hand placement, 95% performed 30 compressions per cycle, 89% performed compressions at a depth of at least 2 inches (5 cm), 90% released after each compression, 97% applied correct breaths, 97% used the automated external defibrillator, and 100% positioned the blades correctly.

The results recorded by the feedback devices used parameters according to the 2010 CPR Guidelines: compression rate (100 compressions per min), compression depth of at least 2 inches (5 cm), and ventilation volumes between 500 to 600 ml. According to the results, 93% used proper hand placement, the average depth of compressions was 48.1 mm, the average release of the chest was 100%, the average number of breaths was 8.2 in 2 min, the average ventilation volume was 742.7 ml, and the percentage of flow fraction was 40.3%. The course was favorably viewed by the participants and specialists.

When students were asked about perceived self-efficacy in performing BLS maneuvers after the online course, 58 (93.5%) expressed confidence in their ability, 2 (3.2%) were not confident, and 2 (3.2%) expressed doubt.

## Discussion

Learning BLS maneuvers is considered highly important. According to the AHA, preparation and qualification for resolution assistance significantly influence the success of resuscitation and increase the chance of survival.^3^ The earlier the course presented to the students, the greater the possibilities of re-training and application in the field of practice, in stages and educational activities on BLS, giving new meaning to the learning and experiences gained by students. Frequent references to the course contributes to learning retention of life support maneuvers, given that knowledge tends to degrade over time.^13^

Three studies on healthcare providers, comparing self-instruction without instructor involvement versus an instructor-led course, demonstrated either no difference or inferior performance during self-instruction.^14^^-^^16^ Feedback devices allowed the participants to evaluate their own performance. These studies emphasized that knowledge on the performance of the maneuvers is very different from effectively carrying out the actions. Moreover, feedback devices can assist in monitoring the procedure, given their accuracy and objectiveness in evaluating student's performance.

The 2015 AHA guidelines state that it is reasonable to use high-fidelity simulators and feedback devices, which will support better CPR quality.^17^ During CPR training, learners who use devices that provide corrective feedback have improved compression rate, depth, and recoil compared to those who do not use such devices.^3^

Three randomized trials examined the use of auditory guidance (i.e., the use of a metronome or music) to guide CPR performance. These studies showed that compression rate was much better when auditory guidance was used, although one study showed a negative impact on compression depth.^18^^-^^20^ If feedback devices are not available, auditory guidance (e.g., metronome or music) may be considered to improve adherence to recommendations for chest compression rate alone. These recommendations are based on balancing the potential benefit of improved CPR performance with the cost of the devices during training. The importance of training frequency is generally accepted. According to the 2015 AHA guidelines, short and frequent trainings are highly recommended because the higher frequency in the study improves the retention and safety during the application of life support.^21^^,^^22^

Although satisfactory levels of theoretical learning are attained after a course or training, retention of life support skills over time tend to decrease.^23–25^ Resuscitation guidelines suggest the use of online courses as resources in life support education. Moreover, they indicate that short periods of study and regularity seem to positively influence learning with no significant correlation with training time.^26^ Therefore, the online course is a resource that can be used for training students and for the continuing education of professionals.^27^^,^^28^

The traditional teaching method, which has long been used to teach skills and promote the acquisition of clinical expertise, is no longer accepted as the best way to teach students. It is necessary to encourage students or professionals to become confident in their abilities. Focusing on the patients’ needs instead of their own is an essential quality of a safe and competent practitioner.^29^^-^^31^

A limitation of this study includes the use of a convenience sample. Furthermore, the strike process and shutdown of the university may have caused the students to be overloaded with activities, making it difficult for them to participate in the research.

## Conclusions

The online course was an effective teaching and learning method, wherein students were able to apply BLS correctly during simulated practice. Furthermore, the online course can be used in continuing education because frequent use of short videos increases learning and self-efficacy during cardiac arrest care. Future research may assess prospects beyond immediate learning using larger samples.

### Acknowledgments

We would like to thank our colleagues from School of Nursing, University of São Paulo (EEUSP) and Emergency Mobile Service (SAMU 192 - SP), who participated in the research.

### Conflict of Interest

The authors declare that they have no conflict of interest.
